# Giant direct and inverse magnetocaloric effect linked to the same forward martensitic transformation

**DOI:** 10.1038/s41598-017-13856-5

**Published:** 2017-10-17

**Authors:** J. I. Pérez-Landazábal, V. Recarte, V. Sánchez-Alarcos, J. J. Beato-López, J. A. Rodríguez-Velamazán, J. Sánchez-Marcos, C. Gómez-Polo, E. Cesari

**Affiliations:** 1 Departamento de Física, Universidad Pública de Navarra, Campus de Arrosadía, 31006 Pamplona, Spain; 2 Institute for Advanced Materials (INAMAT), Universidad Pública de Navarra, Campus de Arrosadía, 31006 Pamplona, Spain; 3 Institut Laue-Langevin, CRG’s D1B - D15, F-38042 Grenoble, France; 4 Departamento de Química Física Aplicada, Facultad de Ciencias, Universidad Autónoma de Madrid, Cantoblanco, 28049 Madrid Spain; 5 Departament de Física, Universitat de les Illes Balears, Ctra. de Valldemossa, km 7.5, E-07122 Palma de Mallorca, Spain

## Abstract

Metamagnetic shape memory alloys have aroused considerable attraction as potential magnetic refrigerants due to the large inverse magnetocaloric effect associated to the magnetic-field-induction of a reverse martensitic transformation (martensite to austenite). In some of these alloys, the austenite phase can be retained on cooling under high magnetic fields, being the retained phase metastable after field removing. Here, we report a giant direct magnetocaloric effect linked to the anomalous forward martensitic transformation (austenite to martensite) that the retained austenite undergoes on heating. Under moderate fields of 10 kOe, an estimated adiabatic temperature change of 9 K has been obtained, which is (in absolute value) almost twice that obtained in the conventional transformation under higher applied fields. The observation of a different sign on the temperature change associated to the same austenite to martensite transformation depending on whether it occurs on heating (retained) or on cooling is attributed to the predominance of the magnetic or the vibrational entropy terms, respectively.

## Introduction

Magnetic shape memory alloys display unique properties associated to the occurrence of a thermoelastic martensitic transformation (MT) between magnetically ordered structural phases. The MT (a reversible first order solid-solid transformation) typically takes place between a high temperature phase (austenite) and a low temperature phase with lower crystalline symmetry (martensite) and distinct magnetic characteristics due to their different crystallographic structures. In particular, in the so-called metamagnetic shape memory alloys, the MT takes place between a ferromagnetic austenite and a weak magnetic martensitic phase^1–3^, so the large magnetization drop taking place at the MT allows the induction of the reverse MT from martensite to austenite by an applied magnetic field^4^. In fact, the magnetic field shifts the transformation temperature, *T*_*m*_, to lower temperatures, stabilizing the high magnetization phase. Magnetic-field-induced martensitic transformations under reasonable magnetic fields have been reported in Ni-Co-Mn-In^4^, Ni-Mn-In^5^, Ni-Mn-Sn^6^, Ni-Co-Mn-Sn^7^, Ni-Mn-Sb^8^, Ni-Co-Mn-Al^9^ and Ni-Co-Mn-Ga^10^ alloys, and the interesting properties related to this effect are being actively investigated because of their potential applications (sensors, actuators, damping, energy harvesting, etc.). Furthermore, since the different magnetic exchange interactions in each of the structural phases result in a discontinuity in the magnetic entropy at the MT, the magnetic induction of the MT may lead to giant magnetocaloric effects in these alloys, which may then be adiabatically cooled by application of a magnetic field. These large magnetocaloric effects make these materials especially interesting for room temperature magnetic refrigeration applications^11–22^.

As a consequence of the counterbalance between the vibrational and the magnetic contributions to the entropy change at the transformation, the forward MT can be inhibited (arrested) on cooling by the application of a strong magnetic field^23–32^, and hence the austenitic phase be stabilized at low temperatures, far below *T*_*m*_. The magnetically retained austenite evolves to martensite as soon as the field is removed or reduced^27^, but, depending on both the temperature and the applied field, a significant (even large) amount of austenite may still persist after the magnetic field removing (apart from an instantaneous partial phase change, the reduction in the applied field leads to a small time-dependent evolution of the transformed fraction^26^). On heating, this metastable austenite transforms to martensite through an ‘anomalous’ forward MT, whose associated magnetocaloric effect has not still been evaluated. In this work, we analyze the characteristics of the retained austenite and determine the entropy change linked to such a peculiar MT in a Ni-Mn-In-Co alloy. We show that the low temperature regime in which it occurs favors the predominance of the magnetic contribution (at the expense of the vibrational one) to the total entropy change, and this leads to the observation of a direct magnetocaloric effect (*ΔS* < *0*), contrary to the inverse effect (*ΔS* > _*0*_) observed in the conventional forward MT. A large adiabatic temperature change of 9 K under a moderate applied field of 10 kOe is reported for the ‘anomalous’ forward MT. This is almost twice the values obtained in the conventional forward transformation under higher applied fields, and one of the largest values obtained in magnetic shape memory alloys.

## Results and Discussion

**Figure 1:**
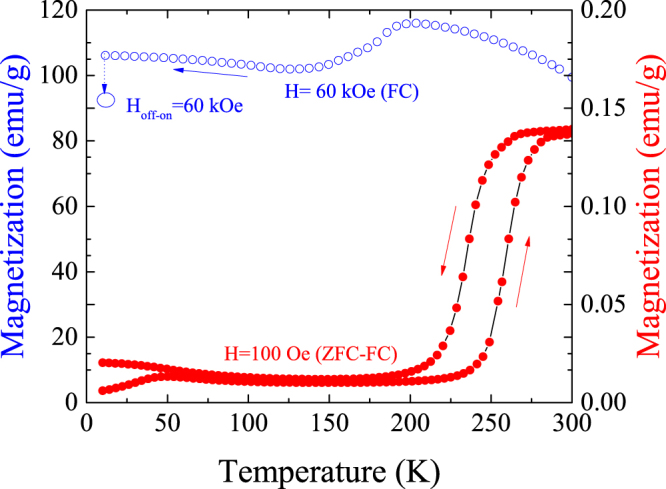
Arrest of the austenite on cooling. Magnetization (*M)* versus temperature (*T*) measured on cooling at 60 kOe and ZFC-ZC curves at 100 Oe.

To kick off, let us describe the magnetization behavior of the Ni-Mn-In-Co alloy. The *ZFC-FC* magnetization measurement at 100 Oe (red dots) in Fig. 1 shows a jump at around 250 K linked to the standard martensitic transformation, as well as a temperature splitting around 50 K corresponding to a spin-glass like behavior already reported^29^. Likewise, the magnetization on cooling under a 60 kOe magnetic field evidences the above mentioned decrease of *T*_*m*_ with the increasing applied field. On the other hand, taking into account that the saturation magnetization of the martensite at 10 K lies around 20 emu/g^27^, the high magnetization value measured at this temperature (~105 emu/g) on cooling under a 60 kOe field indicates the presence of a high quantity of magnetically-retained austenite. The subsequent removal of the magnetic field at 10 K allows a partial transition to martensite, as pointed out by the small decrease in magnetization after switching on again the magnetic field (empty blue dot).

**Figure 2:**
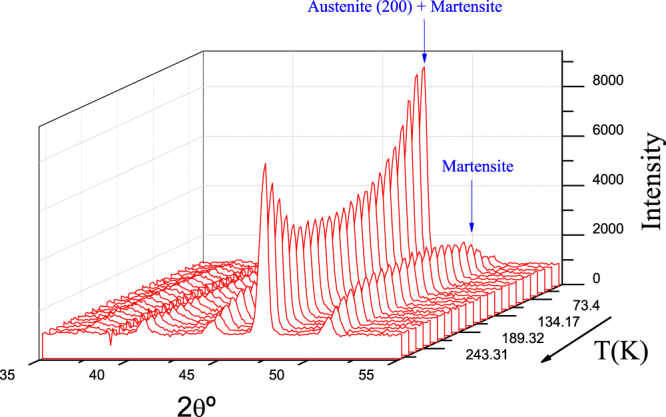
Phase evolution on heating after 70 kOe FC. Neutron thermo-diffraction patterns measured on heating (external magnetic field set to zero at 10 K) in a sample cooled from 300 K under a 50 kOe magnetic field.

A direct evidence of both the austenite retention after field removing and the phase evolution upon subsequent heating is presented in Fig. 2, where we show neutron diffraction measurements performed after field cooling, once the external magnetic field is set to zero at 10 K. In effect, the sample cooled from 300 K under a 50 kOe magnetic field shows a mixture of austenite and martensite at 10 K. The anomalous MT from retained austenite to martensite on heating is evidenced by the reduction of intensity the (200)_A_ austenitic reflection at 2*θ*  =  47.5^°^ (λ = 2.41 Å) and the corresponding increase in the martenstic one around 52^°^. The austenitic peak intensity does not cancel out due to the coincidence of martensitic and austenitic reflections at the same angle. Conversely, as expected, the conventional reverse MT from martensite to austenite takes place on heating above 200 K, where the austenitic peak intensity starts increasing (and the martensite peak intensity starts decreasing).

**Figure 3:**
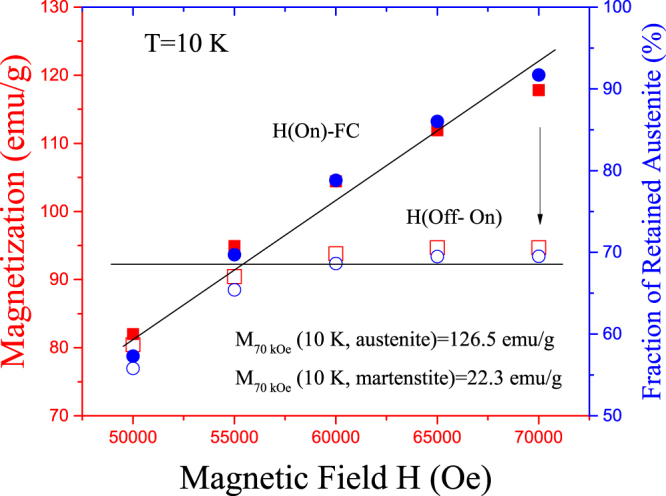
Stability of the retained austenite. Magnetization at 10 K on cooling at different applied fields (full red squares) and after switching off and on the magnetic field (empty red squares). The corresponding fraction of arrested austenite is also plotted in blue circles (right axis).

As shown in Fig. 3, the stability of the retained austenite can be assessed from the magnetization values measured on cooling under different applied fields and the corresponding values after switching off and then on again the magnetic field. In fact, using the values of the saturation magnetization at 10 K for austenite and martensite (126.5 and 22.3 emu/g, respectively^27^), the magnetization changes can be transformed in mass fraction evolutions. Thus, for instance, a sample cooled under 70 kOe retains around 92% of austenite before field reduction, 70% of which remains at zero magnetic field (blue circles, right axis). Similar behavior is found on cooling under different magnetic fields above 55 kOe, with the same fraction value of remaining austenite after field removing (around 70% in all cases). Lower magnetic fields, in turn, are not able to retain such high amount of austenite on cooling, and lower fractions of austenite are observed (i.e. 56% at 50 kOe). Therefore, a 70% of austenite seems to be the upper limit of retained phase at 10 K, below which no evolution is observed after field removing.

**Figure 4:**
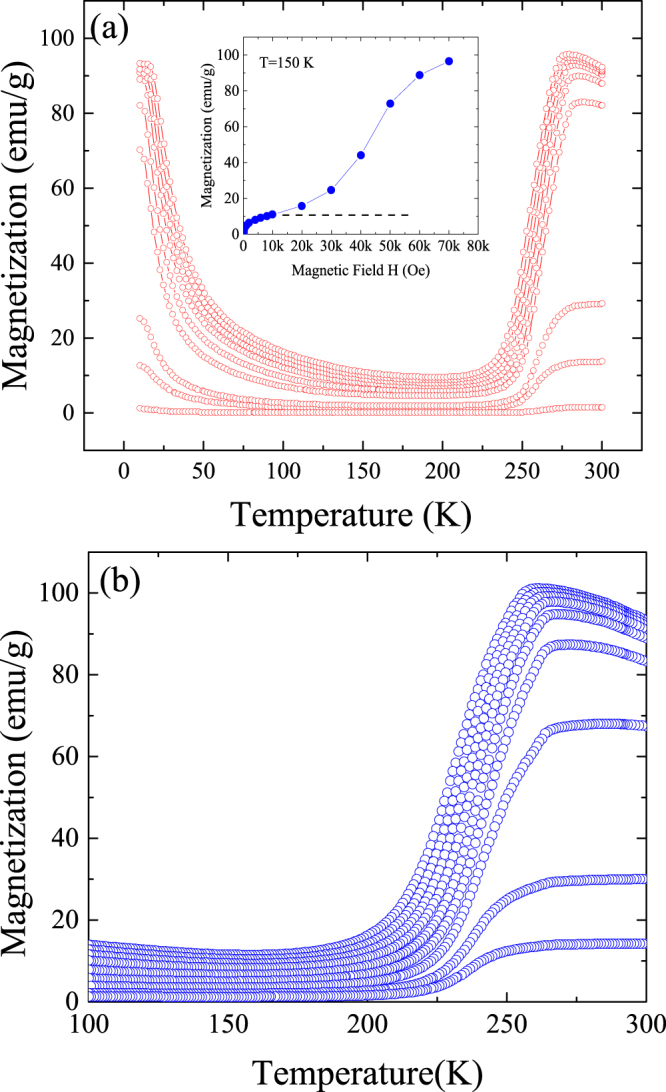
Field dependent thermo-magnetization. (**a,b**) Upper panel: Magnetization *M(T)* at 100, 200, 500, 1000, 2000, 4000, 6000, 80000 and 10000 Oe, measured during heating after Field Cooling under 70 kOe to 10 K. The inset shows the *M(H)* curve at 150 K. Lower panel: Magnetization *M(T)* at 100, 200, 500, 1000, 2000, 4000, 6000, 80000 and 10000 Oe, measured during cooling (forward MT).

From a crystallographic point of view, the structural state of the retained austenite at 10 K under magnetic fields above 55 kOe (70% of austenite) and its change during heating is the same as that occurring at 265 K on *ZFC* during the forward MT. Nevertheless, their intrinsic properties are different because of the different temperature range involved. In particular, the large magnetization of the retained austenite at low temperatures makes it metastable against martensite, which is the stable phase at low temperatures, since the transition to a non-magnetic martensitic phase requires the disordering of the magnetic moments, thus implying an increase in the energy of the system. The transition to martensite must indeed occur on heating once temperature provides that enough energy to destroy the magnetic order. In order to illustrate it, Fig. 4a shows the evolution of the magnetization as a function of temperature *M(T)* under different magnetic fields below 10 kOe (as explained below, higher fields retain some of austenitic phase on heating). The magnetization drop observed on heating above 10 K accounts for the forward retained-austenite to martensite MT, which takes place at higher temperatures on increasing the magnetic field. In any case, only the martensitic phase is present in the alloy at 100 K for those applied fields. The inset in Fig. 4a shows the field dependence of the magnetization at 150 K obtained from the curves shown in Fig. 4a (and those obtained at higher fields, not shown here). Below 10 kOe the magnetization approaches to the saturation value in martensite at 150 K, whereas the large increase in saturation magnetization observed for higher magnetic fields points out to a growing austenite retention. On further heating, the standard reverse MT occurs above 225 K. The magnetization decrease linked to the conventional forward MT taking place on subsequent cooling is shown in Fig. 4b.

**Figure 5:**
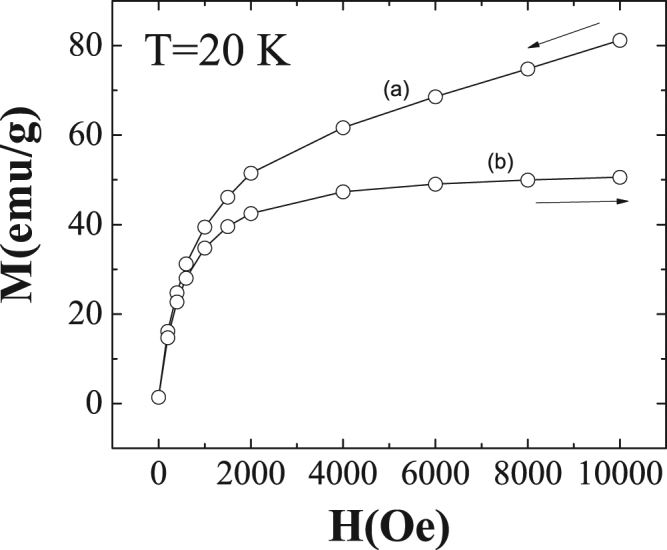
Field dependent magnetization. Magnetization *M(H) measured* at 20 K after a 70 kOe FC to 10 K field removing at 10 K and subsequent heating to 20 K under H = 10 kOe. The magnetization is measured first reducing the magnetic field from 10 kOe (curve **a**) and then increasing the field (**b**).

Briefly, after cooling in a strong enough magnetic field, an anomalous forward MT first occurs on heating (corresponding to 70% of austenite) and then a complete standard reverse MT at higher temperatures follows (Fig. 4a), whereas the standard forward MT occurs on cooling (Fig. 4b). Therefore, the same forward austenite to martensite transformation can be produced by heating and by cooling. The entropy change linked to the different MT sequences and its influence on the refrigeration capacity linked to the magnetocaloric effect can be evaluated from magnetization measurements. Nevertheless, to carefully analyze the thermo-magnetization curves, Fig. 5 shows the field dependence of the magnetization at 20 K (the same qualitative behavior is observed in the 20–50 K temperature range) beginning with the demagnetization curve from 10 kOe and the subsequent magnetization process.

As expected, the initial high magnetization (around 80 emu/g) agrees with the corresponding value of the thermo-magnetization curve in Fig. 4a and the field reduction decreases the magnetization (demagnetization). Nevertheless, the subsequent magnetization reaches a maximum (around 50 emu/g) lower than that found at the beginning. This is a clear evidence of the austenite to martensite transformation occurring during field removing. Once the transformation occurs, the low magnetic field is not able to induce the system to re-transform again back to the initial state as a consequence of the non-reversibility of the transformation.

The magnetocaloric effect is defined as the temperature change *ΔT* produced when a magnetic field is applied or removed under adiabatic condition, or equivalently, as the entropy change *ΔS* in isothermal conditions. In a first approximation, both terms are related through the specific heat (*C*_*p*_) of the sample by^33^

1 $$\Delta T=-(T/{C}_{p})\Delta S$$

According to classical thermodynamics, the magnetic entropy change *ΔS* as a consequence of a magnetic field increase can be indirectly measured by the magnetization curves shown in Fig. 3 and using the following expression^33^,

2 $${\rm{\Delta }}S=S(T,H)-S(T,0)={{\int }_{0}^{H}(\frac{\partial M}{\partial T})}_{H}dH$$

and negative (positive) values of the entropy change are expected when the magnetization decreases (increases) with temperature for the full set of applied fields. In the same way, a magnetic field reduction induces an entropy change $S(T,0)-S(T,H)=-{\rm{\Delta }}S$ (hereinafter *ΔS* will be associated to magnetic filed increases a *−ΔS* to magnetic field reductions).

**Figure 6:**
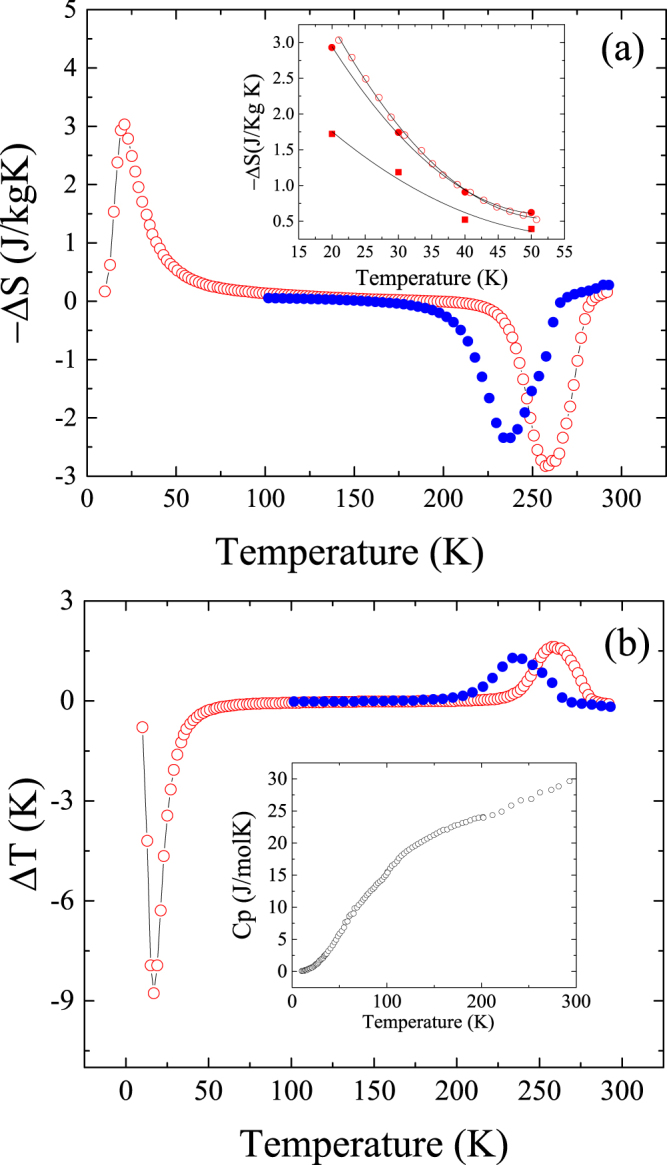
MCE effect as a function of temperature. a, b, (**a**) Entropy change −***Δ*** *S(T)* = *S(T*, *0)* − *S(T*, *H)* at 10 kOe on heating after Field Cooling under 70 kOe (open circles) and during cooling (full circles) with no magnetic field. The inset shows the comparison between the entropy change measured from data in Fig. 4(a) (empty red dots) with that obtained using demagnetization (full red dots) and magnetization (full red squares) M(H) curves. (**b**)Temperature change ***Δ*** *T(T)* under a 10 kOe magnetic field reduction estimated through equation (2). The inset shows the heat capacity measured during heating after a ZFC process.

Due to the irreversibility depicted in Fig. 5, the use of the data in Fig. 4 to calculate the entropy change has only significance for the magnetic field reduction obtained from the initial out of equilibrium state. Under this condition, the field reduction induces the austenite to martensite transformation. The measured entropy change −*ΔS* (field removing) at 10 kOe linked to the ‘anomalous’ forward MT is shown in Fig. 6a at low temperatures. Figure 6a also shows at higher temperatures the entropy change linked to the reverse MT on heating (empty red circles) and to the forward MT on cooling (full blue circles). The ‘anomalous’ MT produces a negative *ΔS* entropy change while both the reverse and the standard forward MT positive values. Focusing on the low temperature transformation, the measured entropy change is the same to that calculated from *M(H)* demagnetization curves in the 20–50 K temperature range (see full red circles in the inset in Fig. 6). The use of the subsequent magnetizations, Fig. 5b, would give the true reversible entropy change, but this value cannot be estimated form data in Fig. 4. The inset in Fig. 6a also shows the entropy change obtained during the magnetization process (red squared symbols). In this case, the entropy changes *ΔS* are half of the values obtained during the demagnetization process but is still negative.

The same austenite to martensite transformation on heating (anomalous) and on cooling (standard) shows opposite sign entropy changes. The total entropy change linked to the MT has mainly vibrational, magnetic and electronic contributions, Δ*S* = Δ_*Svib*_ + Δ_*Smag*_ + Δ_*Selec*_, the electronic one being much lower and therefore usually neglected. During the transformation from paramagnetic martensite to ferromagnetic austenite *∆S*_*vib*_ > *0* and *∆S*_*mag*_ < *0* typically occur^34,35^. In the temperature range of the standard MT, the vibrational contribution is larger and the magnetic-field-induced austenite increases the entropy of the system $(\frac{\partial M}{\partial T} > 0)$. On the contrary, the vibrational entropy of both phases approaches to zero at very low temperatures and therefore the vibrational contribution to the MT is expected to be very small. Furthermore, the lower is the temperature, the higher the magnetic entropy contribution. Thus, in the range where the retained austenite is metastable (below 50 K) the magnetic contribution to the total entropy dominates $(\frac{\partial M}{\partial T} < 0)$ and, accordingly, |*∆S*_*mag*_ | > |*∆S*_*vibr*_|.

Figure 6b shows the temperature change associated to a 10 kOe magnetic field reduction. ∆T has been estimated through expression (1), using the heat capacity (*C*_*p*_) measured during heating (without applied magnetic field) shown in inset in Fig. 6b. To correct the differences (around 10%) between *C*_*p*_ of the austenite and martensite phases^36^, the used capacity was increased by a 7% which corresponds to a 70% of maximum retained austenite. On heating and around the forward anomalous MT negative temperature changes are observed but around the standard forward and reverse MT positive values appear. Interestingly, a remarkable high value of the adiabatic temperature change (~9 K) is observed around the ‘anomalous’ forward MT under a moderate applied field of 10 kOe. This is almost twice that obtained in the conventional forward transformation under higher applied fields (*∆T* ~ 6 K)^22^, and one of the largest values obtained in magnetic shape memory reported both by direct and indirect methods^37^. The large temperature change obtained is simply a consequence of the low specific heat value at low temperatures. The entropy change linked to the Martensitic Transformation is similar at low and high temperatures but the temperature change at low temperature is higher due to the smaller value of the specific heat.

This giant magnetocaloric effect linked to the undercooled austenite phase shows the relevance of the entropy contributions and in particular the outstanding role of the magnetic entropy. In the conventional forward martensitic transformation taking place in metamagnetic shape memory alloys, the magnetic contribution to the total entropy change at the transformation is always counterbalanced by the vibrational one, the latter being always higher. In the limit, both contributions would cancel each other resulting in the absence of transformation. In the present case, in turn, the retention of ferromagnetic austenite and the low temperature regime in which it transforms to martensite favors the, otherwise impossible, predominance of the magnetic contribution to the total entropy change at the transformation, as long as the vibrational entropy of both phases approaches to zero. This leads to the peculiar situation where the sign on the temperature change associated to the austenite to martensite transformation is different depending on whether it occurs on cooling (conventional) or on heating (retained), and highlights the outstanding role of magnetism on driving the structural transformation. From the application point of view, the magnetocaloric effect at low temperatures could compete with other materials like molecular magnetic compounds for magnetic refrigeration at cryogenic temperatures^38,39^, while the occurrence of direct and inverse magnetocaloric effects associated to the same forward martensitic transformation could be useful in the design of refrigeration devices based on more complex thermodynamic processes.

## Methods

A Ni_45_Mn_36.7_In_13.3_Co_5_ alloy was produced by arc-melting followed by several consecutive re-melting in order to homogenize the ingot. After 24 h homogenization at 1170 K under vacuum, samples were annealed at 1070 K for 1800 s and slowly cooled in air to obtain an ordered alloy. Differential Scanning Calorimetry (DSC) analysis reveals that in zero field the martensite to austenite transition peak maximum appears at *A*_*p*_ = 258 K. The transformation entropy, estimated as the latent heat *ΔH* (2.6 J/g) divided by DSC peak temperature, *A*_*p*_, is *ΔS*_*tr*_ = 10 ± 1 J/(kg.K) and the Curie temperature, *T*_*c*_ = 386 K. The heat capacity *C*_*p*_ was measured using a PPMS system. The temperature dependences of the magnetization *M(T)*_*H*_ at different applied magnetic fields were determined using a Quantum Design MPMS XL-7 SQUID magnetometer. The evolution during heating of the arrested austenite in the Ni-Mn-In-Co metamagnetic shape memory alloy has been analyzed by *in situ* neutron diffraction performed in the D20 diffractometer (λ = 2.41 Å) at the Institute Laue-Langevin.
